# Effect of a Surfactant in Microcapsule Synthesis on Self-Healing Behavior of Capsule Embedded Polymeric Films

**DOI:** 10.3390/polym10060675

**Published:** 2018-06-17

**Authors:** Jiyeon Lee, Seon Joo Park, Chul-Soon Park, Oh Seok Kwon, So Young Chung, Jongwon Shim, Chang-Soo Lee, Joonwon Bae

**Affiliations:** 1 Department of Applied Chemistry, Dongduk Women’s University, Seoul 02748, Korea; vito9270@kribb.re.kr (J.L.); moki513@dongduk.ac.kr (S.Y.C.); blueray@dongduk.ac.kr (J.S.); 2 Hazards Monitoring Bionano Research Center, Korea Research Institute of Bioscience & Biotechnology (KRIBB) 125 Gwahak-Ro, Yuseong-Gu, Daejeon 34141, Korea; seonjoopark86@gmail.com (S.J.P.); cspark@kribb.re.kr (C.S.P.); oskwon79@kribb.re.kr (O.S.K.); 3 Nanobiotechnology (Major), University of Science & Technology (UST) 125 Gwahak-Ro, Yuseong-Gu, Daejeon 34141, Korea

**Keywords:** self-healing membrane, microcapsule, surfactant, polyurethane, water treatment

## Abstract

Recently, there has been increased interest in self-healing membranes containing functional microcapsules in relation to challenges involving water treatment membranes. In this study, a self-healing membrane has been prepared by incorporating microcapsules with a polyurethane (PU) shell and a diisocyanate core in a poly(ether sulfone) (PES) membrane. Depending on the characteristics of the microcapsule, to precisely quantify the self-healing behavior and performance of the produced microcapsule embedded membranes, it is important to understand the effect of a used surfactant on microcapsule synthesis. It is noteworthy that mixed surfactants have been employed to control and tailor the size and morphology of microcapsules during the synthetic process, and the surfactant system employed was one of the most dominant parameters for affecting the healing capability of microcapsule embedded membranes. Various techniques including microscopy (optical and electron), thermal analyses (DSC and TGA), and water flux measurements have been employed. This article provides essential and important information for future research into the subtle relation between microcapsule properties with varied synthetic parameters and the self-healing behavior of membrane.

## 1. Introduction

Basically, most natural systems such as human skin and tissue, the organs of plants, and bodies of marine animals inherently possess a “self-healing” mechanism. When a certain type of stimulus is applied to a system, certain kinds of deformation occurs. The presence of a dynamic structure and system, which can react upon the arrival of external stimuli, is one of the most important prerequisites for self-healing [1].

This self-healing mechanism has been popularly introduced into polymeric membranes, which are widely used in water treatment [2]. Because of the limits of polymeric membranes, such as robustness, thin configuration, high fatigue due to an incessant flow of water and materials, and long working time, the membranes can be damaged during operation [2]. Precisely monitoring and fixing the damage sites is very challenging, often consuming a lot of time and resources. Moreover, the replacement of an entire membrane module is unavoidable. That is, the integrity of membranes becomes an even more significant issue in relation to membrane technology. Accordingly, diverse self-healing strategies have been introduced to address this problem [3,4,5,6,7,8].

Microcapsule embedded self-healing membranes have become an impressive approach, among numerous chemical and physical approaches, because they are capable of healing damage without intervention [9,10,11,12]. The concept has also been widely explored in relation to the production of self-healing materials [13,14,15]. In this approach, microcapsules containing healant are embedded in a polymer matrix. It has been reported that the microcapsules have provided versatile functionalities for diverse purposes, for example polyelectrolyte [16,17,18]. The application of a stimuli to the entire material disrupts the shell of microcapsules and causes it to release the healant that restores damage. This is quite a challenging task, even if the concept seems both straightforward and simple, because the microcapsules must be sufficiently small and stable under harsh conditions such as stress, shear flow, and the occasional existence of toxic chemicals. Therefore, the properties and performances of microcapsules must be tailored and tuned.

To date, extensive research activities into microcapsule embedded self-healing membranes have been reported, although intensive studies regarding the dependence of self-healing behavior on microcapsule properties have been relatively sparse. In this study, an in-depth examination of the relation between microcapsule synthetic conditions and the self-healing behavior and performance of membranes such as water flux has been systematically conducted using diverse measurement techniques [2]. In order to conduct this experiment, PU microcapsules [19,20,21,22,23,24,25,26] were embedded into a matrix membrane. Interfacial polymerization using mixed surfactant systems was employed to synthesize well-tuned PU microcapsules. It is noteworthy that the surfactant system employed was the most dominant parameter for affecting the healing capability of microcapsules. A subtle change in the type and composition of the surfactant could induce a dramatic difference in self-healing behavior and performance. This study provides a chance to observe an important phenomenon that is easily overlooked, and thus provides essential information for the performance improvement of versatile PU microcapsule embedded self-healing membranes.

## 2. Experimental

### 2.1. Materials

Toluene diisocyanate (TDI), methyl diphenyl diisocyanate (MDI), 1,4 butanediol (BD), chlorobenzene (CB), and surfactants such as gum arabic (GA), dodecyltrimethylammonium bromide (DTAB), and sodium dodecyl sulfate (SDS) were purchased from Aldrich and used as received. Dimethylacetamide (DMAc) was also purchased from Aldrich and dried using a common method. Poly(ether sulfone) (Ultrason E6020P) was provided by BASF.

### 2.2. Fabrication of PU Microcapsules

**Figure 1:**
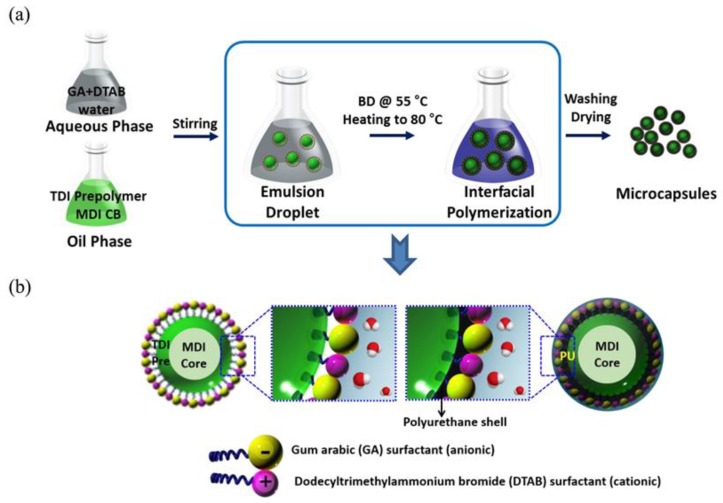
(**a**) The overall synthetic procedure for core-shell type PU microcapsules and (**b**) schematic diagrams of the change in the built emulsion system during reaction.

The overall procedure was very similar to that reported previously [27,28]. The method was based on interfacial polymerization with TDI prepolymer/MDI monomer as an oil phase and surfactants dissolved in water as a continuous phase. The overall synthetic procedure to produce PU based microcapsules is illustrated in Figure 1. The following is the typical synthetic way. A dispersed oil phase was prepared by dissolving home-made TDI prepolymer (3.0 g) in CB (4 mL) by vigorous mechanical stirring and subsequently adding MPI monomer (2 g). Meanwhile, an aqueous phase was prepared by dissolving GA (4.5 g) and DTAB (0.1 g) mixed surfactants in distilled water. The dispersed oil phase was added to the continuous aqueous phase with mild mechanical stirring. The emulsified solution was then heated gradually from room temperature to 55 °C. Next, BD (3.5 mL) was slowly introduced as a chain extender, and the reaction between the TDI prepolymer and BD formed a PU shell. The resultant mixture was further heated to 80 °C and the reaction continued for an additional 0.5 h before completion. The obtained solution was cooled to room temperature naturally. The microcapsules were retrieved after centrifugation, filtering, and drying were repeated more than three times.

### 2.3. Preparation of Microcapsule Embedded Self-Healing Membranes

The membranes were prepared by a well-known solvent-induced phase inversion method [29]. Varying amounts of microcapsules were added in a casting solution containing 10–15 wt % PES in DMAc with vigorous mechanical stirring. The solution containing the microcapsules was cast on an A4 sheet of paper or non-woven fabric using a doctor blade. Then, the obtained film was immediately immersed in a water bath for phase inversion. After overnight immersion, the film was retrieved and dried under ambient conditions.

### 2.4. Instrumentation

The water flux rate of a circular membrane was measured using an Amicon stirred cell (Millipore) pressurized with nitrogen up to 100 kPa [30]. In order to conduct the selective filtration experiment, the surface of a selected membrane was physically damaged using a microtome blade [2]. FT-IR spectra were recorded on a Perkin Elmer Spectrum One spectrometer. Optical microscopy images were taken with an Olympus BX60 microscope. The fluorescence microscope (Olympus, Tokyo, Japan, model IX2-RFA) was utilized for the characterization of the core/shell structure of microcapsules. SEM images were obtained using a JEOL 6700 scanning electron microscopy at an accelerating voltage of 10 KV. Differential scanning colorimeter (DSC) profiles were recorded using a Perkin Elmer DSC 6 at a heating rate of 5 °C/min. Thermogravimetric analysis was conducted using a Perkin Elmer TGA 7 at a heating rate of 10 °C/min.

## 3. Results and Discussion

### 3.1. Microcapsule Properties

To date, extensive study on PU based microcapsules has been conducted, and thus sufficient information regarding such microcapsules is available [27,28]. However, in this article, the effect of a used surfactant system on the properties of microcapsules is investigated using diverse instruments. Two separate solutions were prepared, as depicted in Figure 1a. One was an aqueous solution containing surfactants. Anionic GA was a primary surfactant while cationic DTAB was an auxiliary one. The concentration of added surfactant is far higher than the critical micelle concentration of surfactant system used for this study. A micelle solution was prepared instantly and effectively to produce novel microcapsules [31,32,33]. The other solution was an organic counterpart having precursors for core and shell parts. In this work, polyurethane chemistry was critical and essential both for producing microcapsules and monitoring the effect of surfactants on membrane performance [34]. In this study, aromatic diisocyanate (TDI) prepolymer was employed to form the shell part, since aromatic diisocyanates are more reactive than aliphatic ones as their aromaticity can provide a negative charge to N of the N=C=O group, facilitating the urethane bond formation. In addition, the MDI molecule was selected as a core material, because it has a similar molecular structure as the TDI molecule, but a different relative reactivity.

By mixing the two solutions, an emulsion system, acceptable for the formation of core-shell microcapsules, was built. As BD was added to the emulsion mixture, a dominant reaction between BD and TDI took place, while generated a PU layer surrounding the MDI core (Figure 1b). Even if MDI was also reactive, the facilitated formation of the PU layer could prevent the diffusion of water molecules in the capsules. It was reported that some aliphatic diisocyantes are also eligible to be core materials because they are relatively less reactive than aromatic ones. When defects in the matrix of the shells are disrupted, a potential healing material can be release from the core part.

Some reports found that microcapsule characteristics such as size, size distribution, surface morphology, and stability were dependent on the relative surfactant ratio. Accordingly, we presumed that the surfactant composition (GA/DTAB) would influence the water flux, that is, the self-healing capability, which will be closely examined.

**Figure 2:**
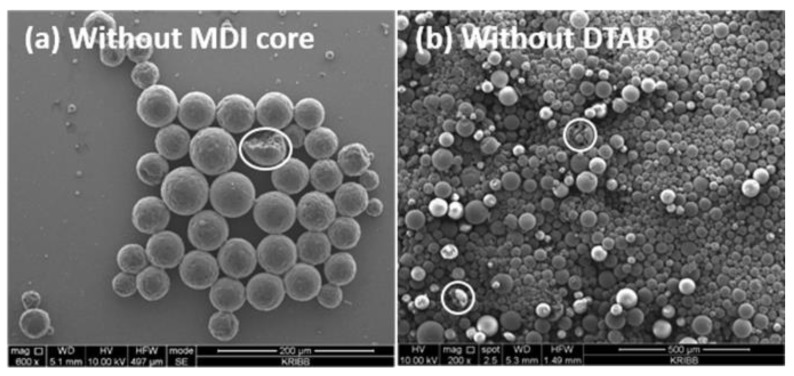
SEM images of PU microcapsules prepared with (**a**) 3.0 g TDI prepolymer, 4.5 g GA, and 0.1 g DTAB without an MDI core and (**b**) 3.0 g TDI prepolymer, 2.0 g MDI, and 4.5 g GA without DTAB.

Typical microscope images of prepared microcapsules produced with 3.0 g TDI prepolymer, 4.5 g GA and 0.1 g DTAB without an MDI core, are shown in Figure 2a. An ample number of microcapsules were successfully prepared by an interfacial polymerization. As can be seen clearly in the SEM image, microsphere-like particles were produced. On the other hand, microcapsules were successfully obtained when the co-surfactant DTAB was not added to the system (Figure 2b). It was almost impossible to discern any morphological variation under this condition. On the contrary, there was an acceptable amount of size distribution because the emulsion polymerization was a thermodynamic equilibrium process easily affected by fluctuations in experimental conditions. It was expected that increasing the concentration of the co-surfactant DTAB would reduce the size variation [35].

**Figure 3:**
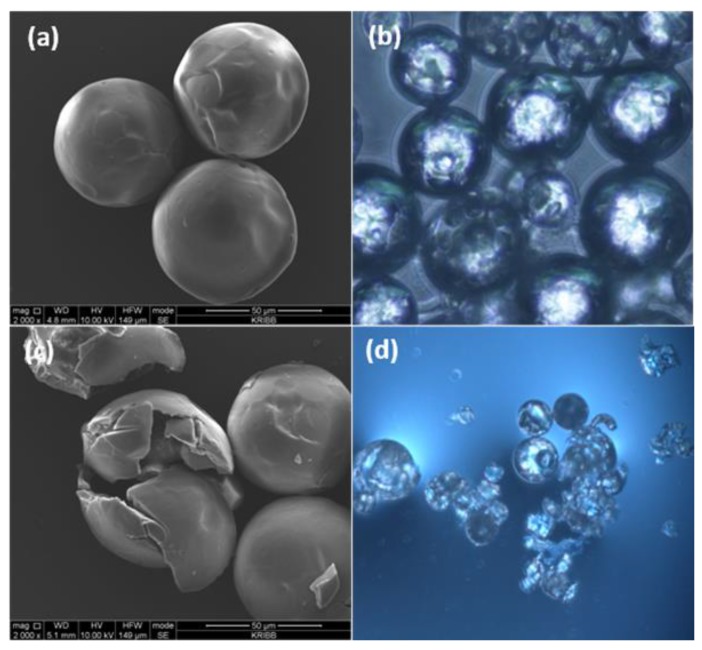
(**a**) SEM and (**b**) OM images of PU microcapsules prepared with 3.0 g TDI prepolymer, 2 g MDI, 4.5 g GA, and 0.1 g DTAB. (**c**) SEM and (**d**) OM images of the same PU microcapsules obtained after manual disruption.

In this study, it was important to prepare microcapsules in the presence of co-surfactant DTAB. SEM and OM images of PU microcapsules prepared with additional 0.1 g DTAB are shown in Figure 3. The appearances of obtained microcapsules were almost identical to those obtained without DTAB. A closer look at the microcapsules revealed that the surface was smooth, homogeneous and continuous (Figure 3a). This image showed that the surface was composed of an identical material, PU. This result was consistent with that obtained by optical microscope monitoring (Figure 3b). A further observation of a disrupted microcapsule, which was obtained after manual disruption, unveiled the hollow inner core of the capsules (Figure 3c,d).

These data are sufficient to demonstrate the appearances of microcapsules. In addition, the core-shell capsule structure was not observed during a supplementary experiment performed with 7.5 g TDI prepolymer (data not shown).

**Figure 4:**
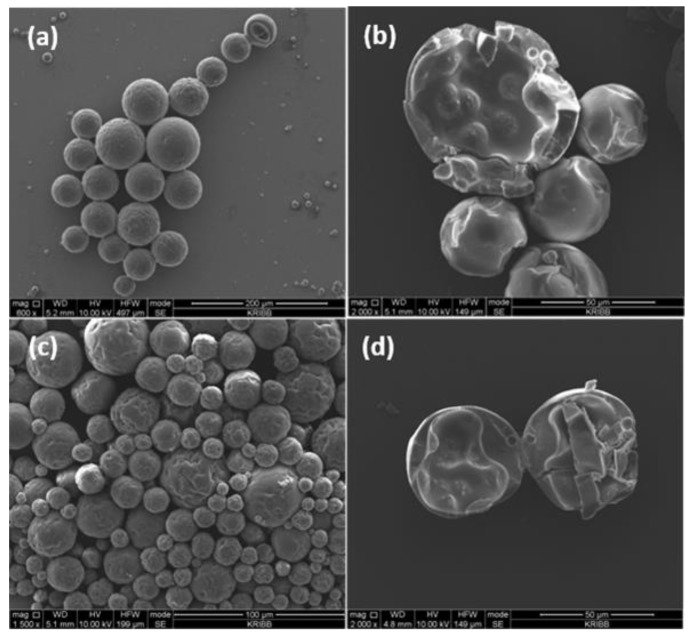
SEM images of PU microcapsules prepared with 3.0 g TDI, 2.0 g MDI, 4.0 g GA, and (**a**) 0.3 g DTAB and (**c**) 0.5 g DTAB. SEM images (**b,d**) of the PU microcapsules obtained after manual disruption of the microcapsules shown in (**a,c**), respectively

A change in microcapsule shape associated with an increase in the DTAB concentration is displayed in Figure 4. As discussed by other research groups [36,37], the average size of microcapsules decreased in general, because all the microcapsules were synthesized by interfacial polymerization. However, a remarkable difference was noticed in the microcapsule shell. As the DTAB amount increased from 0.3 to 0.5 g, wrinkles on the surface of microcapsules conspicuously increased as well (Figure 4a). In particular, a complementary test demonstrated that the wrinkles were more prevalent when 0.5 g DTAB had been used in the synthesis (Figure 4c), which was associated with the following effect. First, the presence of a co-surfactant might cause reactive molecules such as diol or water, to move toward the interfacial region due to the specific interactions that relieve the steric effects between large molecules with opposite charges. This could accelerate the chain extension reaction between the TDI prepolymer and the MDI core material. In this case, the MDI core material was consumed, and residual stress needed to be generated due to the reactions, so that the shells of the microcapsule became crumpled. Therefore, the circular structures protruding from the shells toward the center of microcapsules formed. A closer look at the inner shells of the disrupted microcapsule disclosed this unique property (Figure 4b,d). In addition, it was necessary to consider that the outer shell thickness gradually decreased as the DTAB composition increased, as shown in the images of Figure 3c and Figure 4b,d [37]. Due to the anionic character of the primary surfactant GA, the use of an anionic SDS surfactant instead of DTAB led to undesirable results (data not shown).

**Figure 5:**
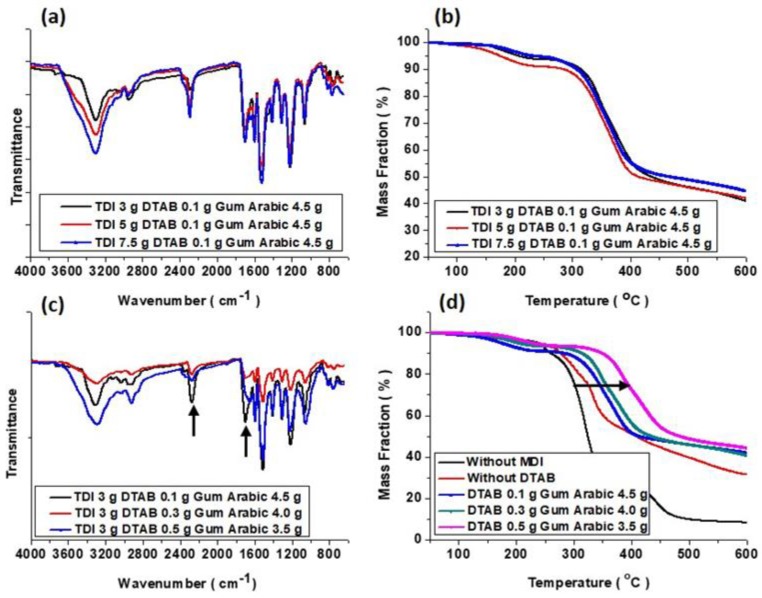
FT-IR spectra (**a**, **c**) and TGA profiles (**b**, **d**) of microcapsules prepared with varying TDI prepolymer and DTAB co-surfactant inputs.

The IR spectra and TGA profiles of microcapsules prepared under various experimental conditions, as shown in Figure 5. The main parameters were the compositions of the TDI prepolymer and DTAB. As indicated in Figure 5a,b, the chemical structure and thermal stability of obtained microcapsules were independent of the TDI prepolymer composition. This tendency was also observed in microcapsules generated from MDI prepolymer. It was acceptable that the molecular structure of MDI is very similar to that of TDI. As shown in Figure 5a, the presence of an N=C=O peak at around 2300 wavenumber indicated the existence of MDI monomer inside the capsules. The depletion of isocyanate groups caused by a reaction to water or amines would make thin peak disappear. The bending peaks at around 800–1500 wavenumber were almost overlapped regardless of the TDI prepolymer input. In addition, the primary thermal degradation temperatures of the capsule shell were identical (Figure 5b). As sufficient amounts of the TDI precursor to shell formation existed, solid shell structures needed to be formed in all cases.

Conversely, a different pattern was discovered when the DTAB input changed from 0 to 0.5 g (Figure 5c). The tendencies in Figure 5c,d indicated that the structure of the microcapsule changed to a different state as the DTAB content was increased. Relative peak intensities of N=C=O and C=O, stretching at around 2300 and 1700 wavenumber, became significantly different to those shown in Figure 5a. The intensity of the peak at around 2300 wavenumber noticeably decreased. In other words, the composition of N=C=O decreased, which meant that the isocyanate was slightly exhausted by the additional reactions, and the PU shell was free from isocyanate groups. It was also evident that the thermal decomposition temperature was fairly elevated (Figure 5d). In any case, a slight improvement in the thermal resistance of the microcapsules implied that the structure of the capsules became relatively robust. These facts indicated that a structural variation in microcapsules occurred due to additional reactions in the residing components such as surfactant complexes, entrapped water molecules, TDI prepolymer and MDI monomer, resulting in a slight increase in the thermal stability of the microcapsule as previously demonstrated in Figure 4.

**Figure 6:**
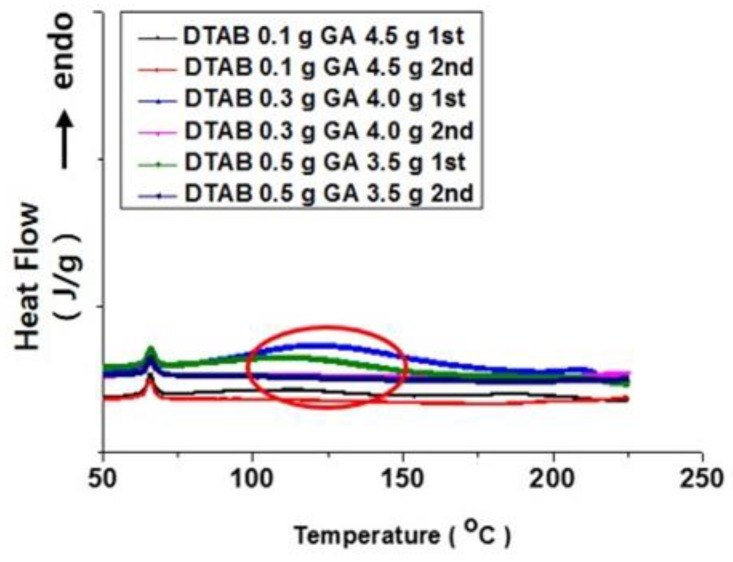
DSC profiles of microcapsules prepared with varying amounts of DTAB.

To support these claims, a DSC analysis was performed for microcapsules prepared with varying amounts of DTAB (0.1–0.5 g) (Figure 6). The profiles clearly indicated that an endothermic peak appeared during the 1st scan for the microcapsules produced with 0.3 and 0.5 g DTAB (see red circle). This showed that residual reaction(s) must have occurred during the 1st scan and disappeared completely after the 1st scan. That is, the baselines of the thermal profiles of all capsules were flat in the 2nd scan. It could be deduced that the reaction(s) were irreversible and endothermic, requiring thermal energy for propagation.

Considering Figure 2 through 5, the increase in the DTAB composition induced a noteworthy effect on the properties and structure of microcapsules. Macroscopically, the fabricated microcapsules with an increased amount of DTAB showed an uneven inner surface and improved thermal stability. A remarkable intensity decrease in the N=C=O stretching peak, at around 2300 wavenumber, implied that the MDI monomer was consumed. At the same time, the increase in the thermal decomposition temperature indicated that the thermal resistance of the capsule shell was improved. These trends must be associated with the DSC profiles in Figure 6. Understanding this phenomenon was important for revealing the relation between microcapsule properties and the self-healing behavior of membranes containing microcapsules.

As the composition of DTAB increased, the electrostatic interactions between GA and DTAB molecules became stronger. In this case, compact micelles could be constructed due to the interactions between opposite charges [35]. The presence of DTAB could attract more BD molecules, because alcohol molecules could be used as a co-surfactant in this system to reduce the steric effect. The polymerization reaction could produce denser microcapsule shells upon the addition of BD, a chain extender. Consequently, the residual reaction between BD and TDI could occur. This aspect could be considered an endothermic peak in DSC profiles. To alleviate the stress induced by the polymerization reaction, the surface of microcapsule shells (in particular, the inner side) became bumpy as shown in Figure 4.

### 3.2. Self-Healing Behavior

**Figure 7:**
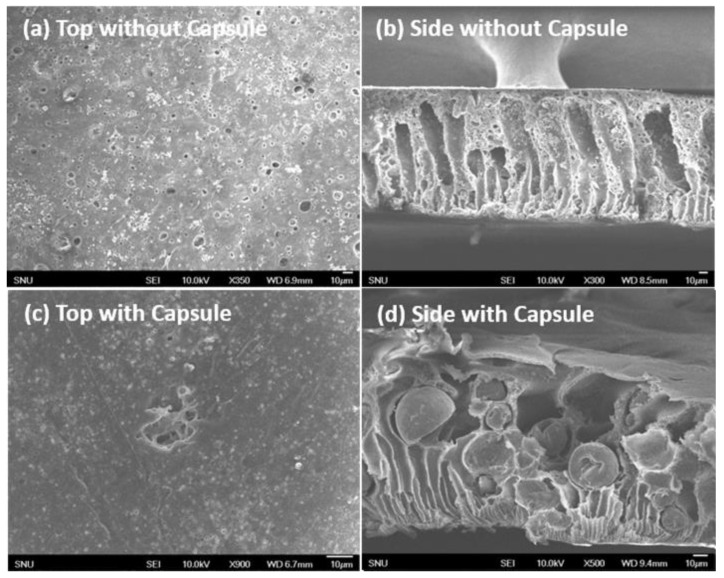
SEM images of pristine (**a**, **b**) and microcapsule (prepared with 3.0 g TDI, 2.0 g MDI, 4.5 g GA and 0.1 g DTAB) embedded (**c**, **d**) PES membranes cast by a phase inversion.

In order to examine self-healing behavior, the produced microcapsules were embedded into a PES polymer matrix. The typical SEM images of a microcapsule embedded membrane (10 wt %) are shown in Figure 7. It was certain that microcapsules effectively resided inside the membrane.

This outcome was desirable, because there was no external stimulus to agitate the structure. In a strict sense, entropy driven migration of microcapsules was unavoidable because the size of the microcapsules was tens of micrometers. If this had happened, there would have been potential damage to the top air/membrane interface. However, this situation did not often arise in our system, as shown in Figure 7.

**Figure 8:**
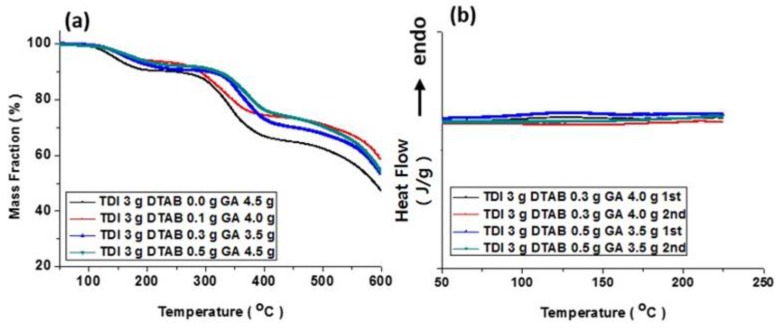
TGA (**a**) and DSC (**b**) profiles for PES membranes containing microcapsules produced with varying DTAB amount.

It was also necessary to confirm that the properties of fabricated microcapsules could be retained inside membranes. Therefore, additional thermal analyses of microcapsule embedded membranes were conducted. The TGA and DSC profiles are shown in Figure 8. It was clear that the trends were also discernable in the membranes containing capsules. The thermal decomposition temperature also increased and, in addition, traces for residual reaction(s) were observed in the DSC profiles. However, the intensity of the peaks of the reaction(s) became weak, because the weight fraction of the microcapsules in the membranes was relatively low. This phenomenon could be acceptable because PES is a very stable engineering plastic.

**Figure 9:**
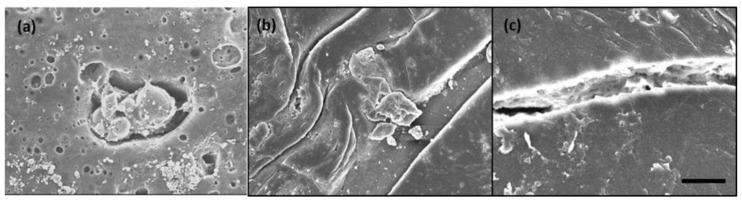
SEM image (**a**) showing the release of core material in the PES membrane. SEM image of PES/PU microcapsule with line damage prepared with (**b**) 0.1 g and (**c**) 0.5 g DTAB after the healing process. (Scale bar is 20 micrometer).

Typical SEM images of membranes containing microcapsules prepared with varying amounts of DTAB, applied after the healing process, are displayed in Figure 9. It was acceptable that the defect sites were filled with a newly formed material, probably derived from an MDI precursor (Figure 9a). A remarkable difference was observed when the microcapsules prepared with different amount of DTAB were employed in the self-healing operation. In the case of microcapsules produced with 0.1 g DTAB, the physical damage was significantly repaired, as indicated in Figure 9b. On the contrary, it was discernible that the defect site was not completely repaired in the membrane with microcapsules prepared with 0.5 g DTAB (Figure 9c).

**Figure 10:**
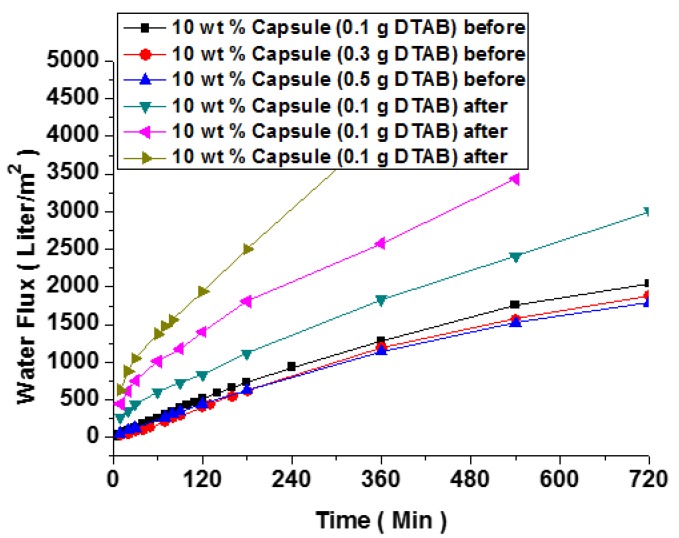
Water flux of membranes with microcapsules obtained before and after the damage and healing process.

To demonstrate the self-healing behavior of microcapsule (10 wt %) embedded membranes, the water flux of sample membranes was measured and is presented in Figure 10. The use of excessive amounts of microcapsules might cause a mixing problem, sharp increase in viscosity and variation in intrinsic properties [2]. The water flux of membranes containing microcapsules was linearly proportional to the exposure time. The water flux of the pristine PES membrane was also measured and found to be similar or slightly lower compared with the values in Figure 10. The presence of microcapsules increased the physical obstruction of flow through the membrane, while providing more internal free volume. To further monitor the self-healing property, the membranes were damaged and healed under water overnight. The introduction of damage rapidly increased the water flux, which was almost restored to its original value in the membrane containing obtained microcapsules prepared with 0.1 g DTAB (black line in Figure 10b). In the membrane containing obtained microcapsules with a larger amount of DTAB, the water flux recovery was retarded from the early stage of healing. It seemed impossible to regain the original water flux value in this sample. In the membrane containing microcapsules prepared with 0.5 g DTAB, the effectiveness of microcapsule breakage upon the stimulus deceased due to the formation of denser and more robust shell, rendering a substantial recovery unachievable.

**Figure 11:**
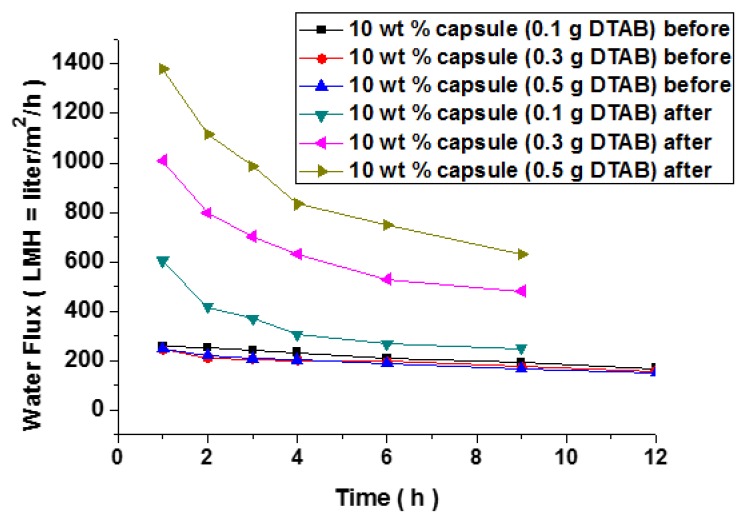
Variation of water flux in LMH unit calculated before and after damage and healing process.

For eye-friendly comparison, variation of water flux values in LMH unit is additionally presented in Figure 11. It is discernable that the variation pattern of water flux was almost similar and consistent before damage. The addition of microcapsules reduced the water flux as shown in the early stage (see square). On the other hand, it can be inferred from Figure 11 that recovery of water flux after healing was achieved for all cases. However, the degree of recovery was retarded with increasing the DTAB content. To further confirm the trend, measurement of the microparticle rejection rate was desirable. We saw that the overall trend in rejection rate was consistent with that seen in the water flux measurement. A detailed study is still underway.

## 4. Conclusions

In this study, poly(ethersulfone) membranes containing polyurethane microcapsules were produced and characterized extensively using diverse methods to investigate the effect of microcapsule synthetic parameters on the self-healing behavior of water treatment membranes. As the amount of auxiliary surfactant (DTAB) increased gradually, the structure of obtained microcapsules became peculiar and, at the same time, the microcapsule shells became denser and more robust. On the other hand, the water flux recovery, intimately associated with the self-healing capability of the microcapsule embedded membranes, significantly deteriorated as the DTAB composition increased. This study is a solid demonstration of the subtle interplay between microcapsule synthetic parameters and membrane property-performance. A closer examination to precisely understand the structure-property-performance relationship in microcapsule embedded polymer membranes is desirable.
